# DU-Net-L: an effective and lightweight segmentation model for alfalfa images that integrates the strengths of DeepLabV3+ and U-Net

**DOI:** 10.1007/s42994-025-00235-2

**Published:** 2025-08-22

**Authors:** Wei Tian, Kang Chong, Jingyu Zhang

**Affiliations:** 1 State Key Laboratory of Forage Breeding-by-Design and Utilization, Institute of Botany, Chinese Academy of Sciences, Beijing, 100093 China; 2 University of Chinese Academy of Sciences, Beijing, 100049 China; 3 China National Botanical Garden, Beijing, 100093 China; 4 Academician Workstation of Agricultural High-tech Industrial Area of the Yellow River Delta, National Center of Technology Innovation for Comprehensive Utilization of Saline-Alkali Land, Dongying, 257300 China

**Keywords:** Alfalfa, DeepLabV3+, U-Net, DU-Net-L, Semantic segmentation

## Abstract

Alfalfa (*Medicago sativa*), a globally important crop known for its high yields, wide adaptability, and high protein content, provides an excellent feed source for livestock. Smart breeding, an emerging technology that integrates genomics and phenomics, holds considerable promise for accelerating the development of elite varieties of alfalfa. Nevertheless, there are few phenotypic analysis tools available for alfalfa. Here, we present DU-Net-L, an effective and lightweight model for segmenting alfalfa images that enables preliminary analysis of branch phenotypes based on digital images. In our study, the DeepLabV3+ model struggled to handle petioles, while U-Net performed poorly with images captured under high light. To address these issues, we have created a new model utilizing ResNet34 as its feature extraction module and retaining the architectures of both DeepLabV3+ and U-Net. An analysis based on test data indicated that the new model overcame the shortcomings of using either of the two base models individually. Subsequently, we lightened the fused model by reducing output channels in each block, while maintaining its predictive capability. We have named the lightened model DU-Net-L. Ultimately, we adopted an exponential decay strategy for the learning rate and increased the number of training epochs to select an optimal parameter combination. This approach achieved 99.83% accuracy and a mean intersection over union of 0.9411, with a size of 25.42 MB. In summary, we have provided a lightweight model that effectively segments stems and leaves in alfalfa images, fulfilling the requirements for the preliminary analysis of branch phenotypes.

**Supplementary Information:**

The online version contains supplementary material available at 10.1007/s42994-025-00235-2.

## Introduction

Alfalfa is a globally cultivated forage crop renowned for its high yield, strong stress resistance, and rich nutritional value for livestock. It is hailed as the “Queen of Forages” and is often the preferred choice for forage cultivation. As a result of economic development, the proportion of meat, eggs, and dairy products in our diets has increased greatly, leading to a growing demand for high-quality alfalfa forage in the livestock industry. Taking China as an example, the import of alfalfa hay has increased dramatically to meet market demand, from approximately 0.2 million to 1.4 million tons between 2010 and 2020 (Wang and Zhang 2023).

Alfalfa exhibits polyploid inheritance, self-incompatibility, and inbreeding depression—characteristics that complicate the discovery of the molecular mechanisms underlying its important agronomic traits. Smart breeding is a strategy that relies on technologies such as whole-genome sequencing and image recognition-based phenomics to accelerate the development of elite crop varieties. By integrating genetic variation data with various types of omics data to establish machine learning prediction models at the whole-genome level (Xu et al. 2022a), it provides design schemes for the efficient and targeted cultivation of new varieties.

This transformative technology represents a key trend in agricultural development, and it offers exciting possibilities for accelerating alfalfa breeding. In recent years, high-quality reference genomes of varieties such as ‘Xinjiang Daye’, ‘Zhongmu No. 1’, and ‘Zhongmu No. 4’ have been successively produced and annotated (Chen et al. 2020; Long et al. 2022; Shen et al. 2020), laying a solid foundation for creating and cultivating improved varieties. Phenotyping platforms for crops such as rice (*Oryza sativa*), maize (*Zea mays*), and wheat (*Triticum aestivum*) have now matured, driving improvements in trait identification and selection (Wang et al. 2023; Yang et al. 2014; Zhang et al. 2024b). However, phenomic analysis tools designed specifically for alfalfa are rare.

Alfalfa is a perennial herbaceous plant that typically grows to a height of 30 to 100 cm. Its stems are upright and clustered, with leaves that are pinnately trifoliate and mostly oblong in shape. Owing to the complex morphology of alfalfa, manual measurements of its traits are time-consuming, labor-intensive, and prone to errors. This greatly hinders both the rapid collection of phenotypic data and the subsequent identification of high-quality molecular markers for a particular trait.

With the development of deep learning technology, it has become feasible to leverage neural networks to quickly and accurately extract plant phenotypes from digital images. Recently, researchers have begun to classify the root system architecture of alfalfa using both traditional machine learning methods and deep learning approaches, achieving satisfactory accuracy rates (Weihs et al. 2024; Xu et al. 2022b). These studies are excellent examples of the benefits of applying phenomics to machine-based phenotypic analysis of alfalfa. Compared to underground parts, the aboveground parts of alfalfa have a more direct link to forage yield, with easier phenotypic data acquisition and a wider range of known agronomic traits. However, specialized tools for analyzing these aboveground traits are lacking. A recent study used deep neural networks to detect alfalfa plants and utilized traditional machine learning approaches for stem counting in proximal images (Bahrami et al. 2025). However, the vast majority of phenotypic data collection for aboveground plant parts remains dependent on manual labor. For example, to assess leaf-related phenotypes, experimenters need to manually measure each individual leaf (He et al. 2025; Mei et al. 2025), an undoubtedly time-consuming and tedious process. Moreover, the measurement process often involves destruction of tissues and organs. For instance, when measuring stem-to-leaf ratios as dry weights, stems and leaves need to be separated, dried, and weighed to obtain the desired data (Lorenzo et al. 2020; Zhang et al. 2024a). With the integration of deep learning technology and plant breeding, it is inevitable that more phenomics methods will be employed in this field to characterize alfalfa growth and development, accelerating the breeding of improved varieties.

Semantic image segmentation is a machine learning process that assigns labels to each pixel in a digital image, allowing for detailed analysis of visual information. DeepLabV3+ is an advanced semantic segmentation model that combines encoder–decoder architecture, atrous convolution, and spatial pyramid pooling techniques (Chen et al. 2018). This model efficiently captures multi-scale contextual information and restores spatial details, achieving high-accuracy segmentation results. A modified version using ShuffleNetV2 (Liu et al. 2022) to extract features and add a convolutional block attention module (CBAM) (Woo et al. 2018) accurately detects picking points of safflowers (*Carthamus tinctorius*), thereby facilitating robotic harvesting (Xing et al. 2024). In addition, DeepLabV3+ has been used to efficiently assess disease severity in wheat plants infected with stripe rust (Liu et al. 2024).

U-Net, named for its distinctive U-shaped architecture, is a lightweight semantic segmentation model initially used for the segmentation of cell images (Ronneberger et al. 2015). Despite its simple structure, it excels at identifying small targets and achieves high accuracy, even with a limited amount of training data. For plant phenotyping analysis, U-Net and its derivative models have been applied to tasks such as the segmentation of individual Chinese cabbage (*Brassica rapa* L. ssp. *Pekinensis*) plants, wheat heads, lesions on tomato (*Solanum lycopersicum*) leaves, and cotton (*Gossypium hirsutum*) roots (Deng et al. 2023; Najafian et al. 2023; Yu et al. 2023; Zhang et al. 2022). Through specific strategies such as performing data augmentation and adding attention layers, these U-Net-based models have achieved accurate semantic segmentation results.

In this study, we focused on the precise semantic segmentation of alfalfa images to develop a network for preliminarily segregating pixels from stems and leaves. We fused the DeepLabV3+ and U-Net models, utilizing ResNet34 (He et al. 2016) as the shared encoder structure for both models, thereby overcoming the limitations of using either model alone. Ultimately, we have created an efficient, lightweight model, which we have termed DU-Net-L, for providing high-accuracy semantic segmentation of stems and leaves in alfalfa images, thereby facilitating machine-based phenotypic analysis for crop improvement.

## Results

### Comparison of basic deep learning models for image segmentation

**Fig. 1:**
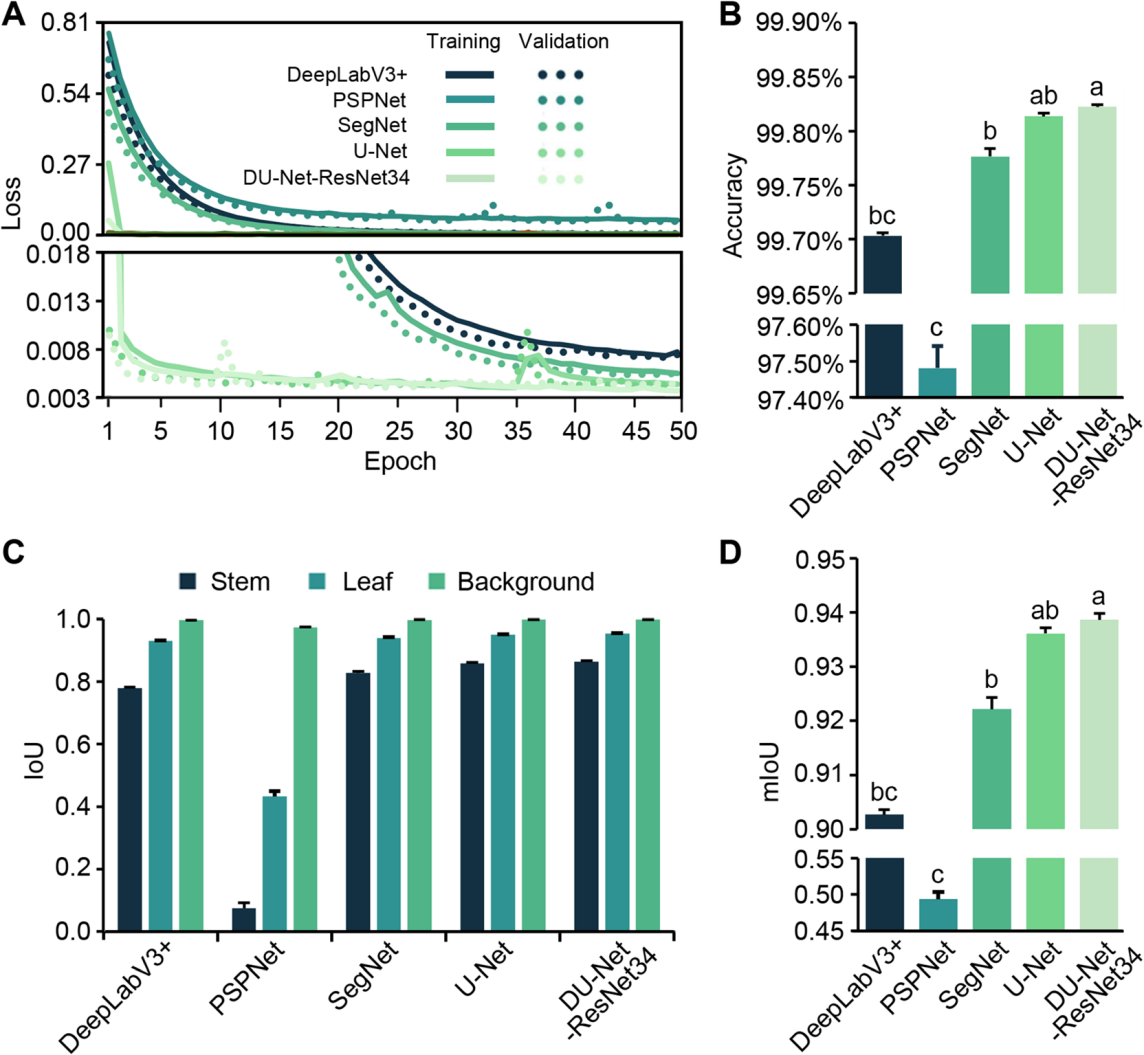
Performance comparison of basic and fused image segmentation models. **A** Training and validation loss. The repetition with the highest accuracy for each model is presented. **B** Accuracy of each model. **C** IoU values for the stem, leaf, and background. **D** mIoU values. All training processes were performed five times. The five epochs with the highest accuracy from each repetition were used for statistical analysis. Error bars indicate + SD of 25 parameter sets. Different letters above the columns in bar graphs indicate significant differences at the *P* < 0.05 level

An overview of the experimental pipeline used for phenotypic analysis is shown in Fig. S1A. Initially, we conducted performance comparisons using four widely recognized and high-performing deep learning models in the field of image segmentation, to determine the most suitable solution for our specific application in alfalfa. These four models are: DeepLabV3+ (Chen et al. 2018), PSPNet (Zhao et al. 2017), SegNet (Badrinarayanan et al. 2017), and U-Net (Ronneberger et al. 2015). The first two models utilized ResNet34 as the feature extraction module and the auxiliary loss of PSPNet was abandoned in this work. In this study, the loss values for the four models stabilized after approximately 20 epochs of training. Among the models, U-Net exhibited the strongest learning capability, with its loss value converging rapidly in the early stages of training. SegNet slightly outperformed DeepLabV3+, with both models displaying comparable trends in loss reduction, while PSPNet lagged noticeably in this task (Fig. 1A). Although all four models achieved accuracies exceeding 97.50% (Fig. 1B, Table 1), performance varied greatly regarding the organ-specific Intersection over Union (IoU), with a consistent pattern of background IoU > leaf IoU > stem IoU (Fig. 1C). The primary reason for these results might be the pronounced data imbalance in the training dataset, which caused the models to be biased toward the background class.

**Table 1:** Performance comparison among models

Model	Accuracy (%)	mIoU	Model size (MB)	Total mult-adds^a^ (TB)
DeepLabV3+	99.71	0.9049	94.29	0.23
PSPNet	97.56	0.5030	90.12	0.18
SegNet	99.79	0.9257	99.48	1.07
U-Net	99.82	0.9377	138.05	2.45
DU-Net-L	99.83	0.9411	25.42	0.34

^a^Total Mult-Adds refer to the cumulative count of multiplication and addition operations, serving as a critical metric for evaluating a model’s computational complexity

**Fig. 2:**
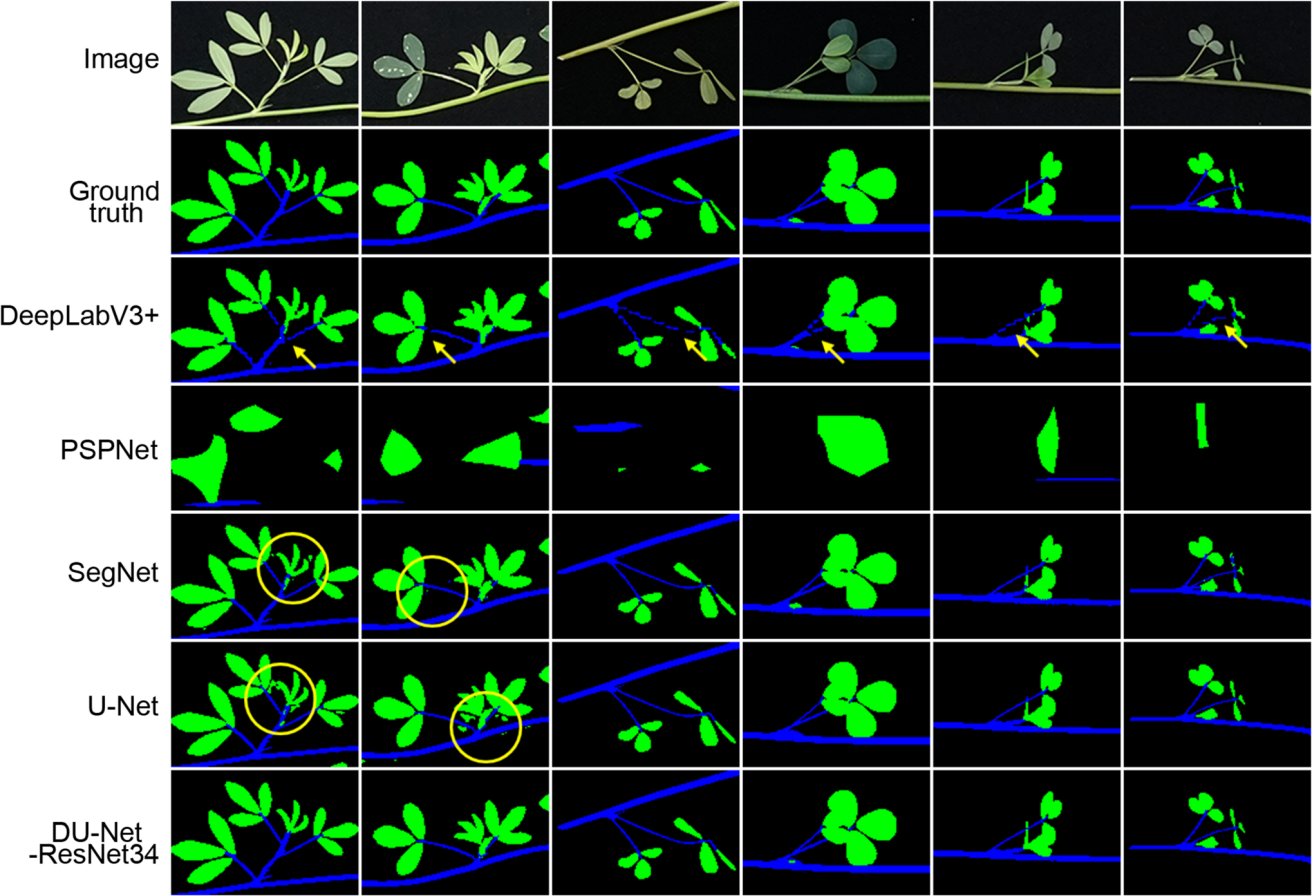
Comparison of prediction results using the four reported models and DU-Net. The predicted breakpoints on petioles are indicated by yellow arrows, while incorrectly predicted spots between leaves are marked by yellow circles. The parameter sets with the highest accuracy for each model were used for the predictions

Upon examining the semantic segmentation results using the test data, we found that DeepLabV3+, SegNet, and U-Net effectively distinguished between the stem and leaf structures of alfalfa branches, whereas PSPNet performed poorly (Fig. 2). PSPNet could only roughly delineate the leaf areas and was barely able to distinguish the stem structures. DeepLabV3+ exhibited deficiencies in accurately resolving the fine structures of alfalfa, notably its inability to predict the petiole effectively. In DeepLabV3+ ’s predictions, all test images displayed discontinuous petiole structures with multiple breakpoints. In contrast, SegNet and U-Net avoided this flaw by accurately predicting intact petiole structures.

However, both models incorrectly classified some background pixels, which were scattered among leaves, as leaf pixels, creating numerous spots around the leaves. This issue was not common; it occurred in only three or four of the 50 images in the test set, all of which were taken in high-light environments. We then gathered another 50 photos taken under high-light conditions and assessed the performance of the models (Fig. S2). The results revealed that the aforementioned problem was observed in 12 SegNet and 8 U-Net predictions, whereas DeepLabV3+ remained unaffected. These results indicate that SegNet and U-Net underperformed when processing images taken under high-light conditions. The similarity in performance of SegNet and U-Net may be attributed to their use of similar U-shaped model architectures. In summary, the performance of PSPNet in this task was unsatisfactory, while all three of the other models, despite their respective limitations, were capable of predicting alfalfa stem and leaf structures.

### Construction of a fusion model

**Fig. 3:**
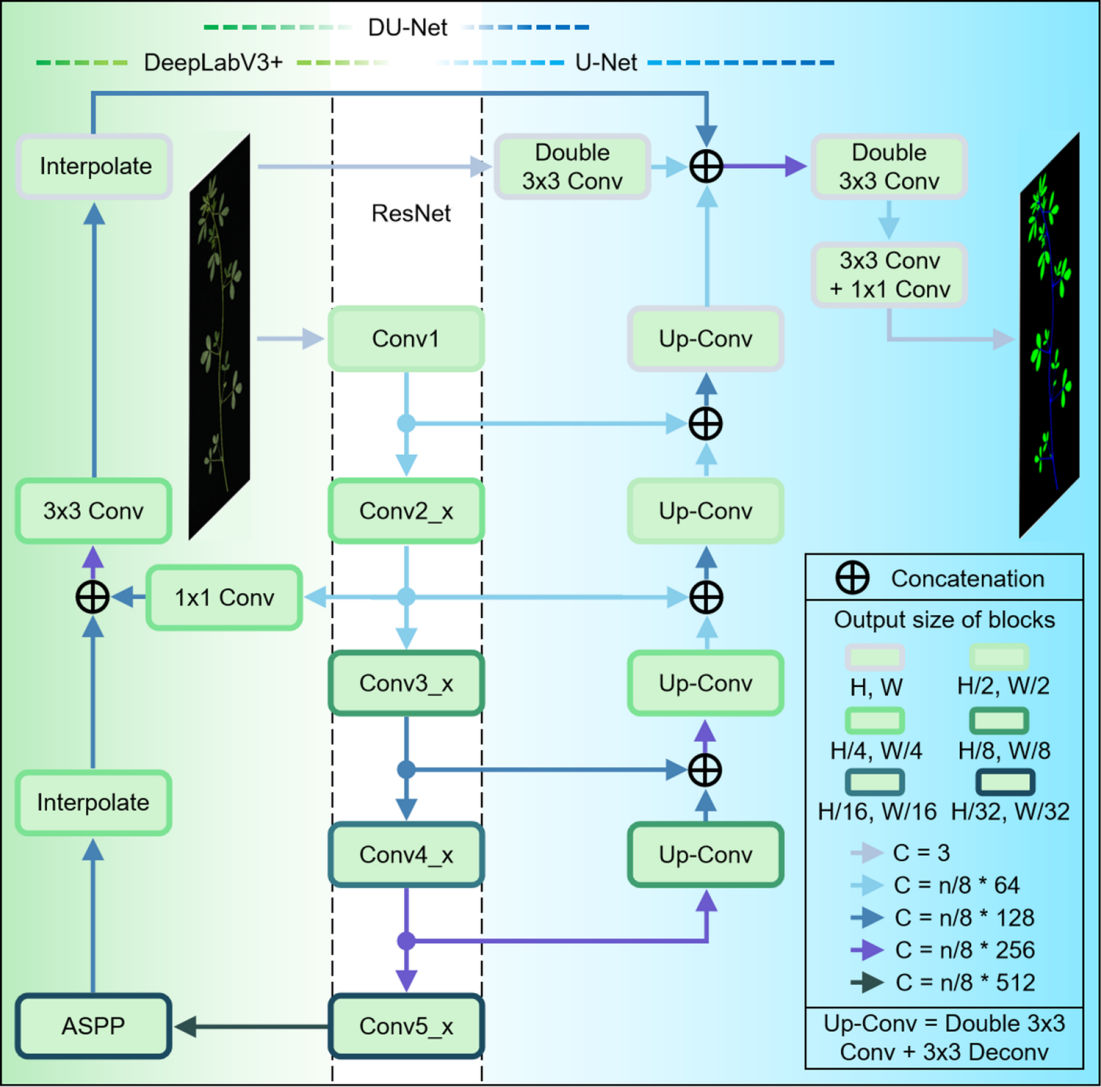
Illustration of the DU-Net structure. The section on the left, with a gradient of green as the background, depicts the unique structure of DeepLabV3+, while the section on the right, with a gradient of blue as background, illustrates the unique structure of U-Net. The middle section with a white background is the ResNet structure, which serves as the shared feature extractor for both DeepLabV3+ and U-Net. H, W, and C represent the height, width, and number of channels of the output feature, respectively, and *n* is an integer ranging from 2 to 8

As noted in the previous section, we found that DeepLabV3+ encountered difficulties in predicting the petiole structure, and U-Net struggled to effectively process images taken under high-light conditions. The observation that these models had different shortcomings inspired us to consider the possibility of fusing the structures of these two models to mitigate the deficiencies of each model when used individually. To test this hypothesis, we integrated the structures of DeepLabV3+ and U-Net (Fig. 3). In our fused model, we employed ResNet34 as the feature extraction module and retained the entire architecture of DeepLabV3+. Building on this architecture, we incorporated the following elements of the U-Net architecture: (1) At the topmost layer, we introduced two 3 × 3 convolutional layers that preserved the input dimensions of height and width. (2) Following the output layers of ResNet34, with the exception of Conv5_x, we added feature concatenating and upsampling layers. (3) Finally, we concatenated the output features from each component and processed them further by convolution to segment the stems and leaves in the input alfalfa image. We named this fusion model incorporating the characteristics of DeepLabV3+ and U-Net as DU-Net, and designated the DU-Net model employing ResNet34 for feature extraction as DU-Net-ResNet34.

We assessed the DU-Net-ResNet34 model using the same evaluation methods as those used above for the previously reported models. Our findings revealed that DU-Net-ResNet34 exhibited a much more rapid convergence of the loss value than U-Net (Fig. 1A). Furthermore, DU-Net-ResNet34 significantly surpassed all other models in both accuracy and mean IoU (mIoU) (Fig. 1B, D). Remarkably, the fusion model overcame the limitations of DeepLabV3+ and displayed sufficient resolving capability for petioles. Additionally, it outperformed U-Net in accurately processing images captured under high-light conditions (Fig. 2, Fig. S2).

### Improvement of DU-Net

**Fig. 4:**
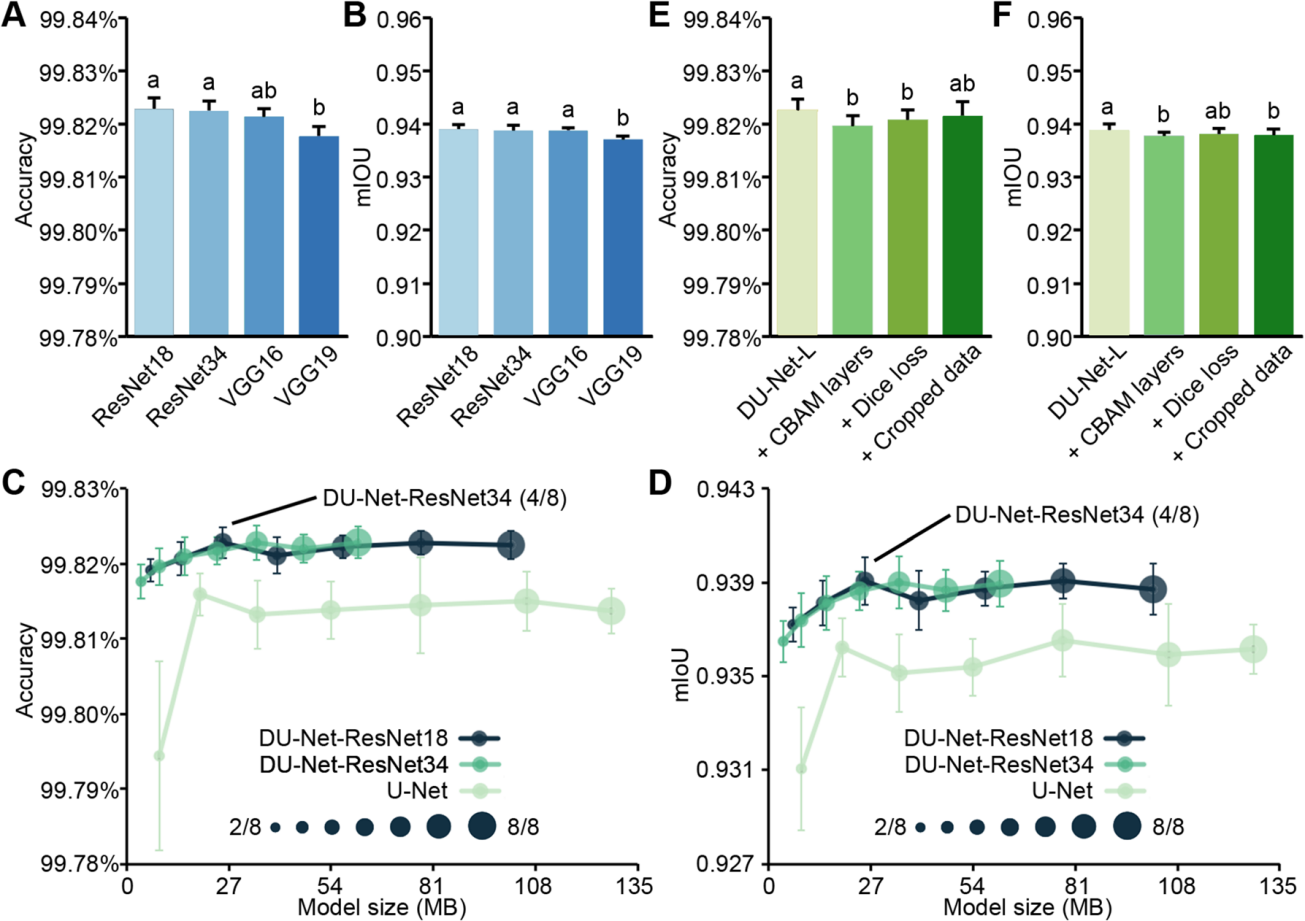
Performance comparison of the improved models. **A**, **B** Accuracy and mIoU of DU-Net models with different feature extraction modules. **C**, **D** Accuracy and mIoU of lightweight DU-Net and U-Net as a function of model size. Ratios like 2/8 denote the proportion of output channels in the lightweight models relative to their original models. **E**, **F** Accuracy and mIoU of further enhanced DU-Net-L. Enhancements were: + CBAM layers, created by adding a CBAM layer after each layer of ResNet34; + Dice loss, which incorporated Dice loss with cross-entropy loss; + Cropped data, which added cropped images to the training dataset. All training processes were performed five times. The five epochs with the highest accuracy from each repetition were used for statistical analysis. Error bars indicate + SD or ± SD of 25 parameter sets. Different letters above the columns in bar graphs indicate significant differences at the *P* < 0.05 level

In the work described above, ResNet34 served as the feature extraction module. Here, we further examined the performance of DU-Net by comparing ResNet18, VGG16, and VGG19 (Simonyan and Zisserman 2014) (Fig. 4A, B). The DU-Net models utilizing ResNet as the feature extraction modules exhibited superior performance compared to those incorporating VGG. Although ResNet34 had a larger parameter size, the DU-Net model with ResNet34 did not outperform the one based on ResNet18.

To minimize the model’s resource consumption and deployment cost, DU-Net models utilizing ResNet were subjected to lightweight processing. We gradually decreased the number of output channels in each block of the fused models by steps of 1/8 of the original count down to 2/8 of that count (Fig. 3). With the U-Net model serving as a control, we assessed the impact of this lightweight adjustment on the performance of these models. Compared to the U-Net model with corresponding output channels, DU-Net models performed better in both accuracy and mIoU (Fig. 4C, D). As the number of output channels was reduced to 2/8 of the original, both DU-Net and U-Net experienced a notable decline in performance; however, the decrease in DU-Net models was less pronounced than that in U-Net model. Notably, the DU-Net model, which utilizes ResNet34 with the number of output channels halved as its feature extraction module, exhibited a low parameter size while achieving an accuracy comparable to that of its counterpart without lightweight processing. Therefore, we selected this lightweight model for further modification, and named it DU-Net-L.

To further enhance the model’s performance, we added a CBAM after each layer of the lightweight ResNet34, aiming to enable the model to better distinguish between stem and leaf structures. However, after adding the CBAM layers, the performance of the model actually deteriorated slightly (Fig. 4E, F). This might be explained by the relatively simple content of our experimental images. The scenario addressed by Woo et al. (2018) requires processing images with diverse backgrounds, whereas our data featured a uniform black backdrop. Moreover, the apical structures of the branches are complex, which may result in less precise annotations for this part of the data. During the learning process, the CBAM layers might have been compromised by these errors, failing to correctly focus on stem and leaf structures. Consequently, the model with the added attention layers ended up performing worse. Given the obvious data imbalance in our training data, we adopted two optimization strategies to address this challenge. The first strategy was to incorporate Dice loss, which is sensitive to foreground regions (such as small objects) and is well-suited for addressing class imbalance issues, in addition to cross-entropy loss. The second strategy was to provide the model with cropped data in which the proportion of background was reduced. Nevertheless, these two enhancements failed to improve the model’s predictive performance; instead, the model’s predictive capability declined slightly after applying these strategies (Fig. 4E, F).

### Determination of optimal parameters

To determine the optimal combination of model parameters, we applied an exponential decay strategy to the learning rate, increased the number of training epochs to 100, and conducted five replicate experiments for each setup. Ultimately, we selected the parameter set that yielded the highest accuracy (99.83%) on the test data, with a corresponding mIoU of 0.9411. As Table 1 indicates, our refined DU-Net-L not only exhibited higher predictive capability, but also featured a significantly smaller parameter size than other models. The computational complexity of the DU-Net-L model is much lower than that of SegNet and U-Net. Although it is slightly higher than that of DeepLabV3+ and PSPNet, the marked improvement in predictive capability it offers makes this trade-off more than worthwhile.

**Fig. 5:**
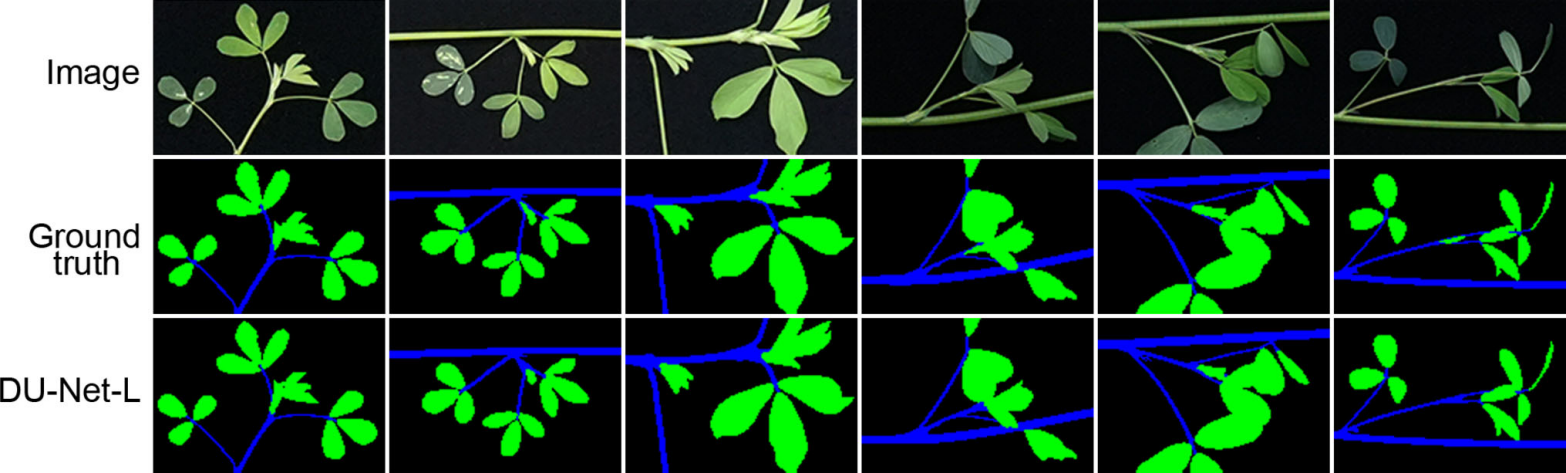
Prediction results of DU-Net-L with fine-tuned parameters

Finally, we evaluated the performance of the optimal model by examining the semantic segmentation results (Fig. 5). The prediction results indicated that our DU-Net-L model was capable of precisely segmenting the stem and leaf structures in alfalfa images. The overall predictions of our model matched well with the manually annotated labels, although discrepancies can be observed in the regions of leaf buds and young leaves. This phenomenon might have two explanations. First, the intricate structures in these regions inherently make it challenging to distinguish between stem and leaf structures, even during manual annotation. Second, the resizing process of the input images may have introduced distortions in the fine details of the labeled images. We then examined the performance of the optimal model on the image set captured under high-light conditions. There were no instances in which background pixels between leaves were misclassified as leaf pixels in the prediction results (Fig. S2). Overall, our fusion model effectively differentiated between stem and leaf structures, fulfilling the requirements for preliminary extraction of branch phenotypes in alfalfa.

## Discussion

In this study, we investigated the application of deep learning models for segmenting the stems and leaves in images of alfalfa plants. Our main conclusions are as follows: (1) DeepLabV3+ faces challenges in effectively handling petiole structures, whereas U-Net encounters difficulties when processing images taken under high-light conditions; and (2) DU-Net-L, which combines the architectures of both models, successfully overcomes the limitations of using either base model alone.

Compared to the reported models tested in this paper, the DU-Net-L model we ultimately created exhibited high accuracy, while also having a small number of parameters and consuming relatively low computational resources. This model effectively accomplished the task of stem and leaf segmentation in alfalfa images, providing valuable insights for image-based alfalfa phenomics. However, the shortcomings of our work are also evident. The most common application scenario of our model is to output leaf-to-stem ratio data from alfalfa. For images with unprocessed branches due to occlusion or other issues, the output stem-to-leaf ratio is a relatively crude result. For images with manually unfolded leaves, the stem-to-leaf ratio data output by the model are highly accurate. Moreover, the semantic segmentation results derived from manually processed branches can be further utilized for the extraction of other phenotypic data, such as leaf length, leaf width, and leaf area. But the process of manually handling the plants demands additional time and effort. To address this issue, it will be necessary to construct a generative adversarial network (GAN) model that can generate pseudo-images of fully unfolded leaves from unprocessed images. The pseudo-images carry accurate phenotypic information about the original branches, thereby eliminating the need for manual pre-processing. GAN is a type of deep learning model that generates high-quality data through adversarial training, consisting of a generator that produces realistic fake data and a discriminator that distinguishes between real and generated data (Goodfellow et al. 2014). We plan to use images of unprocessed branches as the input for the generator. Furthermore, the synthetic images generated by the generator, along with the manually spread-out images, will be used as input for the discriminator. Through iterative adversarial training, this approach will yield a generator that can produce pseudo-images with fully expanded leaves, accurately representing the actual leaf area in the corresponding images of unprocessed branches.

## Materials and methods

### Image acquisition and annotation

The images of alfalfa (*Medicago sativa*) branches used for training the semantic segmentation task were collected from plants grown both in greenhouses and in fields, with no fixed variety in mid-vegetative stages. Then, 300 images of 150 branches (Fig. S1A) were captured using a Sony Alpha 7 IV camera equipped with a Sony SEL2070G lens. During the photography process, the camera was fixed to a top-down mount, and branches were placed on an absorbent cloth directly beneath the camera lens, maintaining a distance of about 1 m between the camera and the branches (Fig. S1B). Initially, photos were taken of the branches without any manipulation. Afterward, the leaves and other structures were fully opened for another set of photographs. A total of 150 branches were photographed, resulting in 300 images from the two methods (Fig. S1C).

Sequentially, images were cropped using Photoshop CS6 to remove unnecessary parts, resulting in cropped images, each at 1440 × 3072 pixels. Following the cropping process, ‘labelme’ software (Wada 2016) was utilized to annotate the stems and leaves of branches. Each individual leaflet of a trifoliate compound leaf was labeled as “leaf,” while the petioles, leaf sheaths, and stems were labeled as “branch.” At the tips of the branches, where newly emerged leaves had not yet unfolded and were clustered with leaf sheaths, the stem structure was not obvious, making it difficult to annotate precisely. In our study, this area was more likely to be labeled as “leaf”. Ultimately, approximately 12,000 annotations were made. After completing the annotation process, the resulting.json files were converted into.png format images using the labelme2seg.py script integrated within PaddleSeg (Liu et al. 2021). Next, 50 annotated images were used as the test set, while the remaining 250 images were utilized for image augmentation to generate the training and validation sets.

### Image augmentation

In order to enhance model robustness, we employed the open-source library “imgaug” (Jung et al. 2020) to perform image augmentation on the training and validation datasets (Fig. S1D). The augmentation techniques included geometric transformations encompassing horizontal and vertical flipping, scaling and expansion, translation, and rotation, as well as color transformations encompassing brightness adjustment, hue and saturation changes, contrast alteration, addition of Gaussian noise, and application of Gaussian blur. By combining these various techniques, we generated 10 augmented images from each original image, ultimately obtaining a total of 2,500 augmented images. In addition to applying the aforementioned techniques, we also incorporated image cropping to address the data imbalance caused by the relatively low proportion of stem pixels. Using the second strategy, we generated an additional 1,250 augmented images.

### Experimental pipeline

The overview of our experimental design is presented in Fig. S1A. We divided the images into training and validation sets in a 7:3 ratio. Images were resized to 480 × 1024 pixels before being fed into the models. Initially, we evaluated four highly acclaimed semantic segmentation models, namely DeepLabV3+, PSPNet, SegNet, and U-Net, using images generated by the first augmentation strategy to establish the foundational architecture of our model. In our model training process, the batch size was set to 5; Adam was selected as the optimizer, with an initial learning rate of 0.001; cross-entropy loss was used as the loss function; and accuracy and mIoU of the test set were used to evaluate model performance. Because DeepLabV3+ struggled to resolve fine details such as petioles, and U-Net was compromised with images captured under high-light conditions, we fused the two models and named the result DU-Net, a model that inherits the strengths of both DeepLabV3+ and U-Net. A comparison assay revealed that DU-Net models utilizing ResNet as the feature extraction modules exhibited superior performance compared to those incorporating VGG. Furthermore, we lightened the fused model by systematically reducing the number of output channels in each block of ResNet, yielding a model that is not only parameter-efficient but also highly accurate. Subsequently, to further improve the model’s performance, we implemented three optimization strategies: adding CBAM layers, changing the loss function, and incorporating cropped data generated by the second augmentation strategy. Then, we adopted a learning rate decay schedule (with lr_lambda set to 0.95^epoch^) and increased the number of training epochs to 100 to obtain the optimal combination of model parameters. Finally, we developed a deep learning model specifically tailored for the semantic segmentation of stems and leaves in alfalfa images, which we named DU-Net-L.

### Hardware and software

All experiments were conducted on the same platform. The computer, running the Windows 11 operating system, was equipped with an i9-13900 K CPU, 64 GB of RAM, and an RTX 4090 GPU. Software versions used were CUDA Toolkit 12.2.2, cuDNN 8.9.5.29, Python 3.11.5, PyTorch 2.1.0 and torchvision 0.16.0.

### Evaluation metrics

Accuracy and mIoU were used to evaluate the performance of networks. Each model’s prediction for each instance was compared with the true label, and the accuracy was calculated by dividing the number of matches by the total number of instances. The formula for accuracy is shown in Eq. (1):

1 $${\text{Accuracy }} = \, \frac{{\text{Number of correct predictions}}}{{\text{Total number of predictions}}}$$

mIoU was calculated as the average of the IoU values for each class, as shown in Eq. (2), where *TP*_*i*_ represents the true positives for class *i* (i.e., the number of pixels correctly predicted as belonging to class *i*); *FP*_*i*_ represents the false positives for class *i* (i.e., the number of pixels erroneously predicted as belonging to class *i* when they actually belong to another class); and *FN*_*i*_ represents the false negatives for class *i* (i.e., the number of pixels that truly belong to class *i* but were mistakenly predicted as belonging to another class). In this study, there were three classes: branch, leaf, and background.

2 $${\text{mIoU}} = \frac{1}{3}\sum\limits_{i = 1}^{3} {\frac{{TP_{i} }}{{TP_{i} + FP_{i} + FN_{i} }}}$$

### Statistical analysis

All training processes were performed five times. Each repetition consisted of 50 epochs, except for the final fine-tuning step, in which the lambda learning rate schedule was applied. Each of these final repetitions included 100 epochs. The five epochs with the highest accuracy from each repetition were used for statistical analysis. Error bars indicate + SD or ± SD of the 25 parameter sets. The Kruskal–Wallis test, followed by the Dunn test, was used to determine whether the performances of the models were significantly different. Different letters above the columns in bar graphs indicate significant differences at the *P* < 0.05 level.

## Supplementary Information

Below is the link to the electronic supplementary material.

Supplementary file1 (DOCX 1997 kb)

## Data Availability

The relevant dataset and code have been uploaded to Kaggle: https://www.kaggle.com/datasets/wildtype2024/images-of-alfalfa-branches-for-segmentation
