# Supplementary material for: DU-Net-L: an effective and lightweight segmentation model for alfalfa images that integrates the strengths of DeepLabV3+ and U-Net
Source: aBIOTECH. 2025 Aug 22;6(4):763–73. doi: 10.1007/s42994-025-00235-2 (PMC12647467; doi:10.1007/s42994-025-00235-2)
Supplement: Supplementary file 1 — Supplementary file1 (DOCX 1997 kb) [file 42994_2025_235_MOESM1_ESM.docx]

**
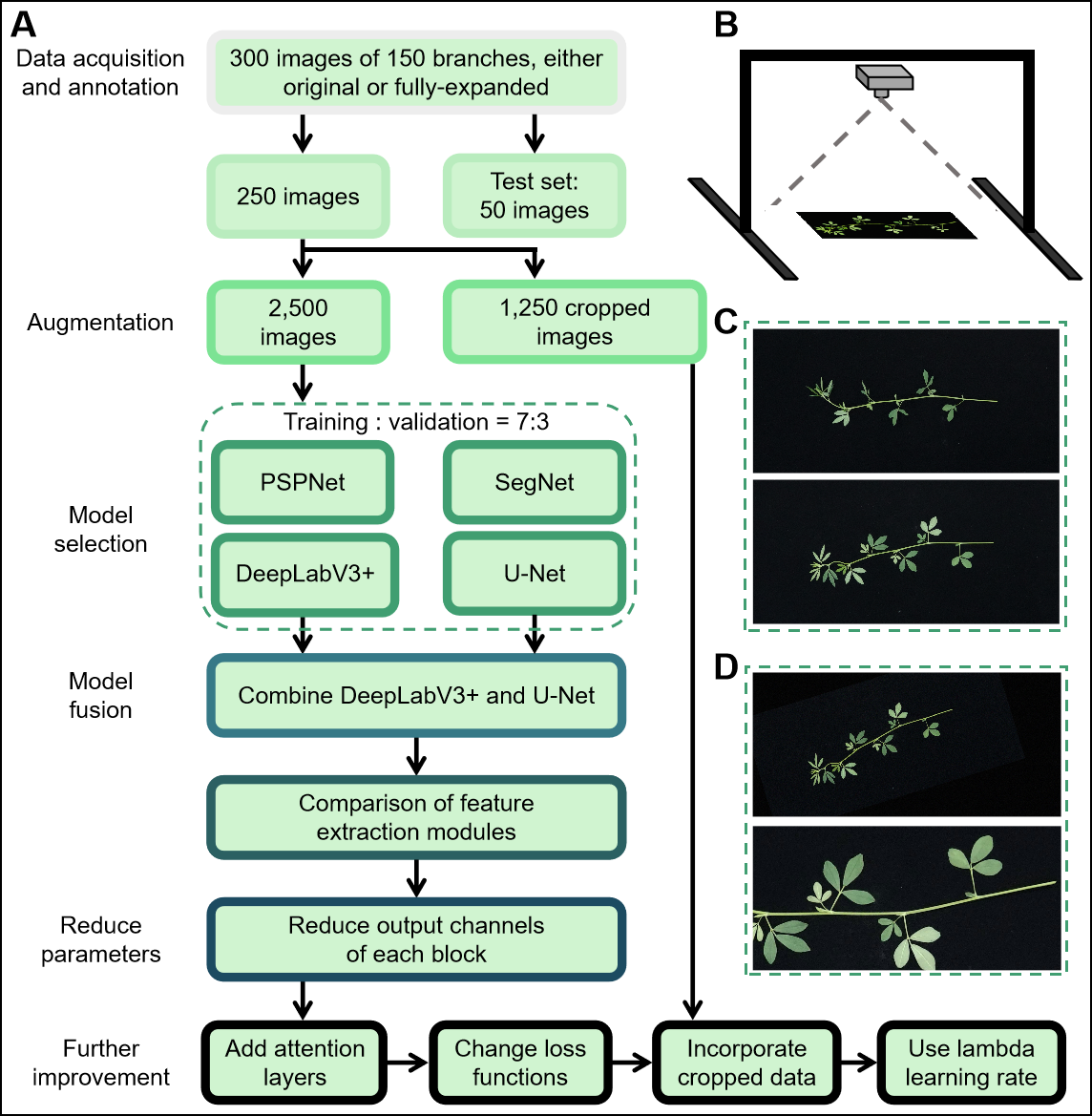
**

**Fig. S1** Experimental pipeline overview. **A** Flowchart of experimental design. **B** Photography method. **C** Images before augmentation. Upper: Image of branch without manipulation; Lower: Image of fully spread-out branch. **D** Augmented images. Upper: Original augmented image; Lower: Augmented image additionally incorporating cropping.


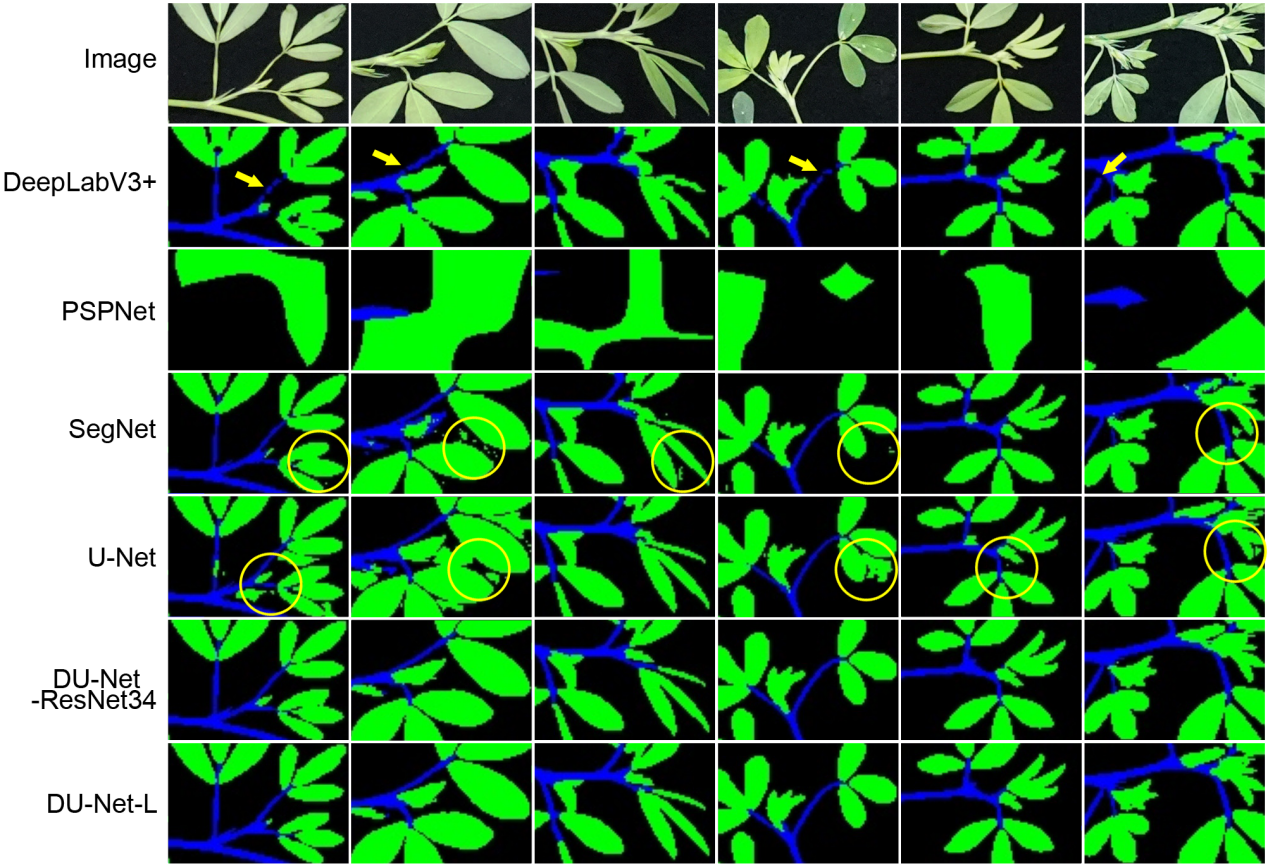


**Fig. S2** Comparison of prediction results for images captured under strong light conditions among reported and DU-Net models. The breakpoints on leaf stalks are indicated by yellow arrows while the incorrectly predicted spots between leaves are marked by yellow circles. The parameter sets that have the highest accuracy for each model are used for the prediction.
